# The Relationship Between Novel Inflammatory Markers and Serum 25‐Hydroxyvitamin D Among US Adults

**DOI:** 10.1002/iid3.70115

**Published:** 2025-01-14

**Authors:** Hang Zhao, Yangyang Zhao, Yini Fang, Weibang Zhou, Wenjing Zhang, Jiecheng Peng

**Affiliations:** ^1^ Department of Cardiology Anqing First People's Hospital of Anhui Medical University Anqing China; ^2^ The Fifth Clinical College of Anhui Medical University Hefei China

**Keywords:** cross‐sectional study, NHANES, novel inflammatory markers, serum 25‐hydroxyvitamin D

## Abstract

**Background:**

Vitamin D is the focus of extensive medical research globally. Recent studies have investigated the correlation between serum 25‐hydroxyvitamin D (25(OH)D) and common inflammatory markers. However, few studies have incorporated novel inflammatory markers such as the platelet‐to‐lymphocyte ratio (PLR), platelet‐to‐high density lipoprotein cholesterol ratio (PHR), systemic inflammatory index (SII), neutrophil‐to‐lymphocyte ratio (NLR), systemic inflammatory response (SIRI), and neutrophil‐to‐high‐density lipoprotein cholesterol ratio (NHR). This study investigated these correlations among adults in the USA.

**Methods:**

We ultimately included a total of 5308 participants from the National Health and Nutrition Examination Survey (NHANES) database spanning 2007 to 2018. A multivariable linear regression model assessed the links between serum 25(OH)D and these novel inflammatory markers, with subgroup analyses for hypertension and diabetes. To further explore the relationship between the two, we applied smooth curve fittings and generalized additive models. Upon detecting nonlinear relationships, we used a recursive algorithm to pinpoint the inflection point.

**Results:**

In our multivariate linear regression model, serum 25(OH)D concentrations were negatively correlated with NHR (*β* = −0.003, 95% CI: −0.005 to −0.001), NLR (*β* = −0.002, 95% CI: −0.003 to 0.000), SII (*β* = −0.579, 95% CI: −0.954 to −0.205), PHR (*β* = −0.171, 95% CI: −0.249 to −0.093), and PLR (*β* = −0.096, 95% CI: −0.051 to −0.040) among adults in the USA. Nevertheless, no significant association was found with SIRI (*β* = −0.001, 95% CI: −0.002 to 0.000). Subgroup analysis by hypertension and diabetes showed that in the hypertensive group, serum 25(OH)D was significantly and negatively associated with NHR, NLR, SII, SIRI, PHR, and PLR. However, no correlation was found in the diabetic group between serum 25(OH)D levels and these inflammatory markers.

**Conclusions:**

Our research confirms that serum 25(OH)D levels are negatively correlated with several novel inflammatory markers among adults in the USA, suggesting potential directions for further research into vitamin D's role in inflammation.

## Graphical abstract

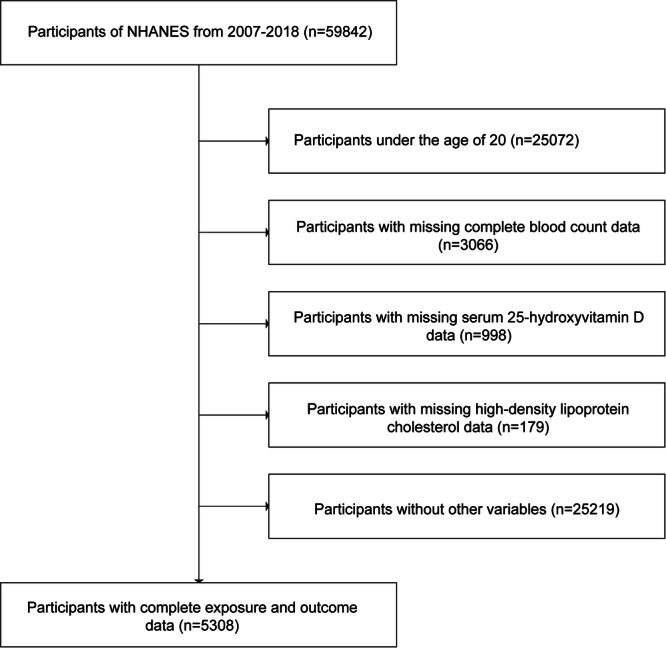

## 1 Introduction

Globally, the increasing recognition of vitamin D status highlights its significance as a critical public health concern [1]. This fat‐soluble vitamin primarily occurs in two variants: cholecalciferol and ergocalciferol. Ergocalciferol is predominantly derived from dietary sources such as cod liver oil, mushrooms, and fish, while cholecalciferol is synthesized in the skin through exposure to ultraviolet rays [2]. Serum 25‐hydroxyvitamin D (25(OH)D) is a bioactive form of vitamin D that remains stable and abundant in the bloodstream; thus, it is the most frequently measured indicator for assessing vitamin D levels [3]. Serum 25(OH)D performs various biological functions within the human body. Despite extensive food fortification efforts, a deficiency in serum 25(OH)D has been identified in 32% of the American population [4]. In recent years, research on serum 25(OH)D has become increasingly prevalent both in the United States and globally, with increasing interest in exploring its physiological roles [5].

Multiple research endeavors have validated the correlation between various inflammatory immune‐related diseases and vitamin D, including metabolic syndrome [5], cardiovascular diseases [6], autoimmune diseases [7], and cancer [8]. Research indicated that vitamin D influences both innate and adaptive immunity [9], primarily by regulating immune cell function, inhibiting the production of inflammatory mediators, modulating inflammatory signaling pathways, promoting the production of immune‐suppressive factors, thus affecting the migration and proliferation of immune cells, and ultimately impacting inflammatory immune‐related diseases [10]. Over the past few decades, clinical and epidemiological research has found the links between serum 25(OH)D and common inflammatory factors, such as fibrinogen or C‐reactive protein. However, a definitive consensus on this relationship has yet to be reached. Oliveira et al. [11] found that CRP was negatively correlated with serum 25(OH)D, which aligns with the findings of a bidirectional mendelian randomization analysis [12] that exploring the relationship and potential causal effects between the two. Additionally, Laird et al. [13] identified a significant relationship between low serum vitamin D levels and specific inflammatory indicators, such as the IL‐6/IL‐10 ratio. Nevertheless, the relationship between inflammatory markers and serum 25(OH)D remains controversial. For instance, a large cross‐sectional study [14] reported no correlation between the two. Similarly, a research with a small sample size by Michos et al. [15] also detected no significant relationship between CRP and vitamin D. This variability introduces a degree of uncertainty into the research field.

In recent years, novel inflammatory factors based on full blood counts, such as PLR, NLR, SIRI, and SII, have been proposed [16]. The immune and inflammatory status of the body can be reflected by these emerging inflammation markers, assisting in the prediction of disease severity and prognosis [17, 18]. In addition, PHR, NHR, and other new inflammation indicators are associated with cardiovascular disease play a crucial role in guiding patient treatment strategies and assessing prognosis. Currently, these novel inflammation factors are widely recognized as accurate indicators of inflammatory status owing to their cost‐effectiveness [19]. Whereas, research on their relationship with serum 25(OH)D concentration remains limited [20]. While previous studies have investigated relationships between vitamin D and common inflammatory factors, few have used the latest inflammatory markers to assess their correlation. Our study aims to fill this gap.

Previous research have explored the relationships between one or two novel inflammatory markers and serum 25(OH)D. Our research is the first to simultaneously investigate the correlations between multiple novel inflammatory markers (NHR, NLR, SII, SIRI, PHR, and PLR) and serum 25(OH)D in the adult population of the United States. This research benefits from a larger sample size compared to previous ones, thanks to the data gathered from the NHANES.

## 2 Materials and Methods

### 2.1 Study Population

**Figure 1:**
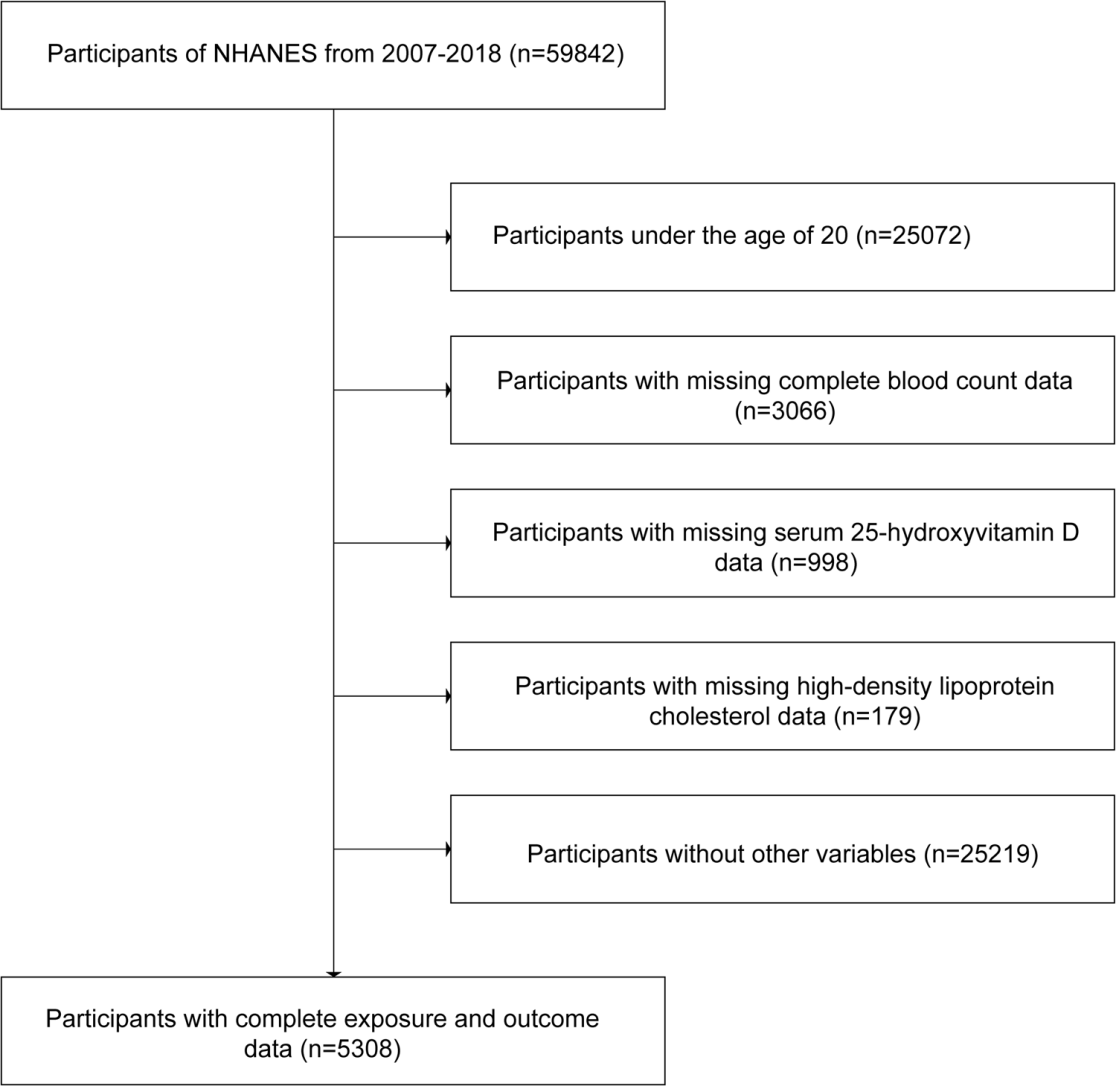
The flowchart of participants.

This research made use of data collected from the NHANES, a survey updated every 2 years. NHANES serves as an ongoing surveillance tool, offering a thorough assessment of the health status and dietary behaviors among the American populace. Our analysis used data from the continuous cycle of the US NHANES data set spanning from 2007 to 2018, throughout the duration of the study, a total of 59,842 participants ranging from 0 to 85 years of age were included. The analysis excluded individuals aged under 20 (*n* = 25,072), those lacking complete blood count with differential data (including neutrophil number, monocyte number, platelet number, lymphocyte number) (*n* = 3066), those missing Serum 25(OH)D data (*n* = 998), those lacking HDL‐C data (*n* = 179), and those with missing data on other variables relevant to the study (*n* = 25,219). Ultimately, the study comprised 5308 participants. The specific steps can be seen in Figure 1, as shown in the analysis. This study is based solely on the publicly available NHANES database, which has been approved by the Research Ethics Review Board [21].

### 2.2 Assessment of Serum 25‐Hydroxyvitamin D, NHR, NLR, SII, SIRI, PHR and PLR

Between 2007 and 2018, blood 25‐hydroxyvitamin D levels were assessed utilizing a standardized liquid chromatography‐tandem mass spectrometry (LC‐MS/MS) methodology. The complete blood cell counts of NHANES participants, including lymphocyte, neutrophil, and platelet counts, were determined using the Beckman Coulter HMX Hematology Analyzer, and reported as ×10^3^ cells/uL [22]. The measurement of HDL‐C levels utilized the Roche Modular P Chemistry Analyzer from 2007 to 2012 and the Roche Cobas 6000 and Roche Modular P Chemistry Analyzer from 2013 to 2018 [23]. The calculation formula for inflammatory markers is as follows (where L represents lymphocyte number, M represents monocyte number, N represents neutrophil number, and P represents platelet number): SII = P*N/L, PHR = P/HDL‐C level, NLR = N/L, PLR = P/L, NHR = N/HDL‐C level, SIRI = M*N/L.

### 2.3 Assessment of Covariates

In this study, novel inflammatory markers (including NHR, NLR, SII, SIRI, PHR and PLR) were considered as outcome variables, while serum 25(OH)D served as the exposure variable. Confounding covariates, such as age, dietary fiber intake, BMI, alcohol intake, protein intake, poverty income ratio, ALT level, AST level, total blood calcium, and triglyceride level, were treated as continuous variables (mean ± SD). Sex, race (Non‐Hispanic Black, Non‐Hispanic White, Hispanic, Other), hypertension (Informed by a doctor or professional medical institution whether diagnosed with hypertension), diabetes (Informed by a doctor or professional medical institution whether diagnosed with diabetes), and smoking status (Everyday, somedays, not at all), were considered categorical variables (%). Extensive data regarding all variables employed in our research is available at www.cdc.gov/nchs/nhanes/.

### 2.4 Statistical Analysis

The statistical analyses were conducted using EmpowerStats (version 2.0) and R software (version 4.0.3) in our research. We defined statistical significance with a P‐value threshold of less than 0.05. For baseline characteristics, categorical variables were analyzed using Chi‐square tests, while continuous variables were assessed through one‐way analysis of variance (ANOVA) or Kruskal‐Wallis tests. To assess the normality of continuous variables, the Kolmogorov‐Smirnov test was applied. The results for continuous variables were presented as means with standard deviations (SD), while categorical variables were expressed as percentages. Considering potential confounding factors, the individual correlations between the inflammatory markers involved in this study and serum 25(OH)D were determined using multivariable linear regression models. Model 1 is a univariable linear regression model, while Models 2 and 3 are multivariable linear regression models. The selection of confounders in the multivariable linear regression models was based on prior knowledge from the literature, clinical relevance, and statistical considerations. Three models were developed as follows: Model 1 remained unadjusted; Model 2 included adjustments for sex, race, and age; and Model 3 included adjustments for age, protein intake, BMI, alcohol intake, poverty income ratio, dietary fiber intake, ALT and AST levels, total blood calcium, triglyceride levels, sex, race, hypertension, diabetes, and smoking status. After identifying a link between serum 25(OH)D and NHR, NLR, SII, SIRI, PHR, and PLR, we conducted subgroup analyses according to the presence of hypertension and diabetes to pinpoint responsive subpopulations. To better understand the association between these inflammatory markers and serum 25(OH)D, we applied smooth curve fittings and generalized additive models. Smooth curve fitting is a nonlinear approach used to depict relationships between variables by fitting a smooth curve, which more effectively highlights trends in the data. Generalized additive models are flexible regression models that capture nonlinear relationships between variables, allowing for complex interactions without assuming that each variable's effect is strictly linear. A recursive algorithm was used to pinpoint the inflection point upon detecting nonlinear relationships. Subsequently, the association was further analyzed using a two‐segment piecewise linear regression model around the identified inflection point. Significance was assigned to P values below 0.05.

## 3 Results

### 3.1 Basic Characteristics

This research comprised 5308 screened participants aged 20–80 years. The baseline demographic and clinical laboratory characteristics of all participants are detailed in Table 1, including race, age, sex, BMI, dietary fiber intake, alcohol intake, protein intake, poverty income ratio, ALT level, AST level, total blood calcium, triglyceride level, hypertension, diabetes, smoking status, and novel inflammatory markers such as NHR, NLR, SII, SIRI, PHR, PLR. Among the participants, 58.53% were males, 51.53% were of White ethnicity, and 18.84% were of Black ethnicity. Significant differences were noted among the different serum 25(OH)D quartiles (Q1 to Q4), except for SII (*p* = 0.507). In the Q4 group, compared to Q1: higher levels of dietary fiber intake, serum total calcium, NLR, poverty income ratio, and PLR were observed; lower levels of BMI, protein intake, alcohol intake, ALT level, NHR, and PHR were noted.

**Table 1:** The characteristics of study participants.

Serum 25(OH)D (nmol/L)	Mean ± SD/*N*%	Q1 (8.32–45.90)	Q2 (46.00–63.10)	Q3 (63.20–81.40)	Q4 (81.50–238.00)	*p* value
**Age (years)**	51.911 ± 17.011	48.749 ± 16.130	49.594 ± 16.916	51.927 ± 16.813	57.334 ± 16.859	< 0.001
**Gender (%)**						< 0.001
Male	58.53	747 (56.548%)	845 (63.870%)	814 (61.065%)	701 (52.667%)	
Female	41.47	574 (43.452%)	478 (36.130%)	519 (38.935%)	630 (47.333%)	
**Race (%)**						< 0.001
Hispanic	21.21	327 (24.754%)	366 (27.664%)	280 (21.005%)	153 (11.495%)	
Non‐Hispanic White	51.53	357 (27.025%)	613 (46.334%)	809 (60.690%)	956 (71.826%)	
Non‐Hispanic Black	18.84	508 (38.456%)	220 (16.629%)	147 (11.028%)	125 (9.391%)	
Others	8.42	129 (9.765%)	124 (9.373%)	97 (7.277%)	97 (7.288%)	
**Poverty income ratio**	2.314 ± 1.585	1.976 ± 1.460	2.193 ± 1.551	2.439 ± 1.621	2.643 ± 1.624	< 0.001
**BMI (kg/m²)**	29.117 ± 6.827	30.385 ± 7.924	29.617 ± 6.767	28.657 ± 6.180	27.823 ± 6.012	< 0.001
**Protein intake (gm)**	82.228 ± 44.811	79.367 ± 48.681	85.813 ± 44.738	82.482 ± 43.209	81.249 ± 42.179	0.002
**Dietary fiber intake (gm)**	15.986 ± 10.401	14.456 ± 10.429	16.830 ± 11.072	16.112 ± 9.969	16.539 ± 9.950	< 0.001
**Alcohol intake (mg)**	13.289 ± 32.657	15.887 ± 38.924	12.303 ± 31.691	12.189 ± 30.569	12.791 ± 28.433	0.010
**ALT (U/L)**	25.533 ± 19.283	26.611 ± 21.545	26.133 ± 18.734	24.011 ± 15.502	25.391 ± 20.726	0.003
**AST (U/L)**	25.944 ± 20.435	26.923 ± 20.668	25.293 ± 25.539	24.587 ± 13.681	26.978 ± 20.053	0.003
**Total calcium (mmol/L)**	2.338 ± 0.088	2.327 ± 0.088	2.331 ± 0.083	2.340 ± 0.086	2.353 ± 0.092	< 0.001
**Triglyceride (mmol/L)**	1.500 ± 1.140	1.484 ± 1.141	1.555 ± 1.200	1.534 ± 1.252	1.426 ± 0.940	0.016
**NLR**	2.265 ± 1.316	2.161 ± 1.184	2.266 ± 1.326	2.280 ± 1.443	2.353 ± 1.292	0.002
**PLR**	126.115 ± 52.545	123.148 ± 54.661	124.256 ± 51.812	128.386 ± 52.045	128.632 ± 51.446	0.010
**SII**	542.174 ± 353.306	530.714 ± 342.521	541.295 ± 361.946	545.808 ± 376.741	550.784 ± 330.241	0.507
**NHR**	3.524 ± 1.987	3.598 ± 2.168	3.762 ± 2.026	3.481 ± 1.929	3.258 ± 1.775	< 0.001
**PHR**	193.165 ± 81.589	200.191 ± 87.114	202.537 ± 79.716	193.194 ± 80.845	176.846 ± 75.931	< 0.001
**Hypertension (%)**						< 0.001
Yes	41.13	535 (40.500%)	480 (36.281%)	535 (40.135%)	633 (47.558%)	
No	58.87	786 (59.500%)	843 (63.719%)	798 (59.865%)	698 (52.442%)	
**Diabetes (%)**						0.030
Yes	15.15	201 (15.216%)	202 (15.268%)	173 (12.978%)	228 (17.130%)	
No	84.85	1120 (84.784%)	1121 (84.732%)	1160 (87.022%)	1103 (82.870%)	
**Smoking status (%)**						< 0.001
Everyday	36.63	605 (45.799%)	501 (37.868%)	441 (33.083%)	397 (29.827%)	
Somedays	8.42	147 (11.128%)	133 (10.053%)	98 (7.352%)	69 (5.184%)	
Not at all	54.95	569 (43.073%)	689 (52.079%)	794 (59.565%)	865 (64.989%)	

*Note:* Continuous variables: Mean ± SD. Categorical variables: *N*%.

### 3.2 Correlations Between Serum 25(OH)D and NHR, NLR, SII, SIRI, PHR, and PLR

**Figure 2:**
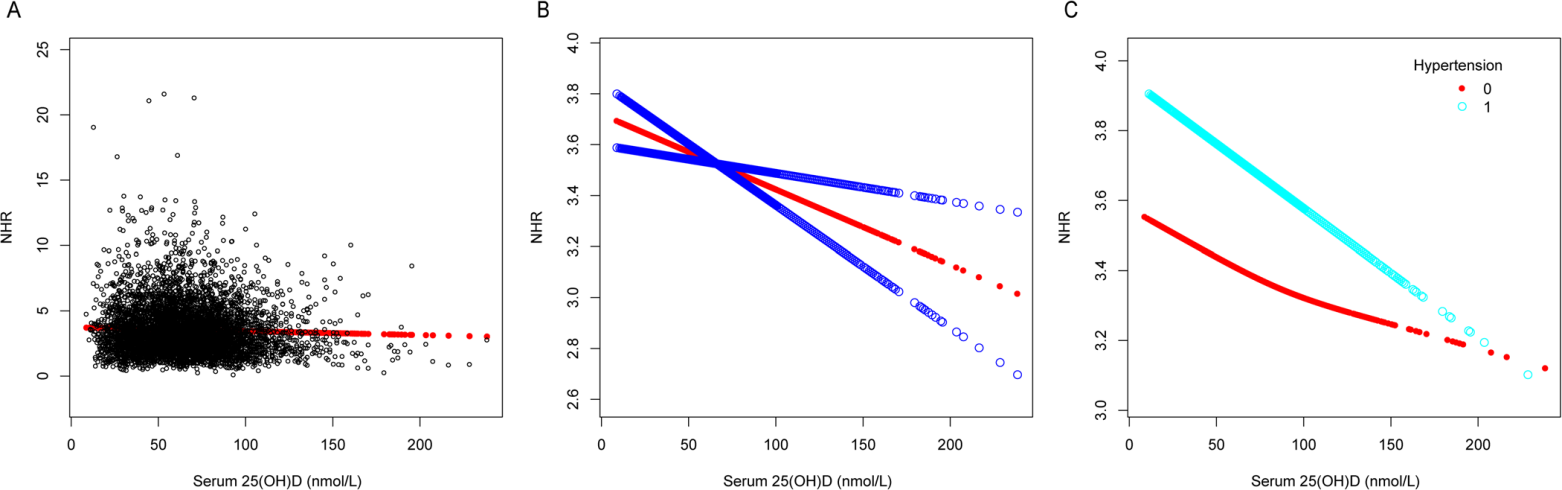
The association between serum 25(OH)D (nmol/L) and NHR. (A) Each black point represents a sample. (B) The smooth curve fit between the variables is represented by a solid red line. The blue bands indicate the 95% confidence interval (CI) of the fit. (C) Stratified by hypertension. The correlation coefficients for (B, C) are shown in the respective figures. Age, protein intake, BMI, alcohol intake, poverty income ratio, dietary fiber intake, ALT and AST levels, total blood calcium, triglyceride levels, sex, race, hypertension, diabetes, and smoking status were adjusted ([C] was not adjusted by hypertension).

**Figure 3:**
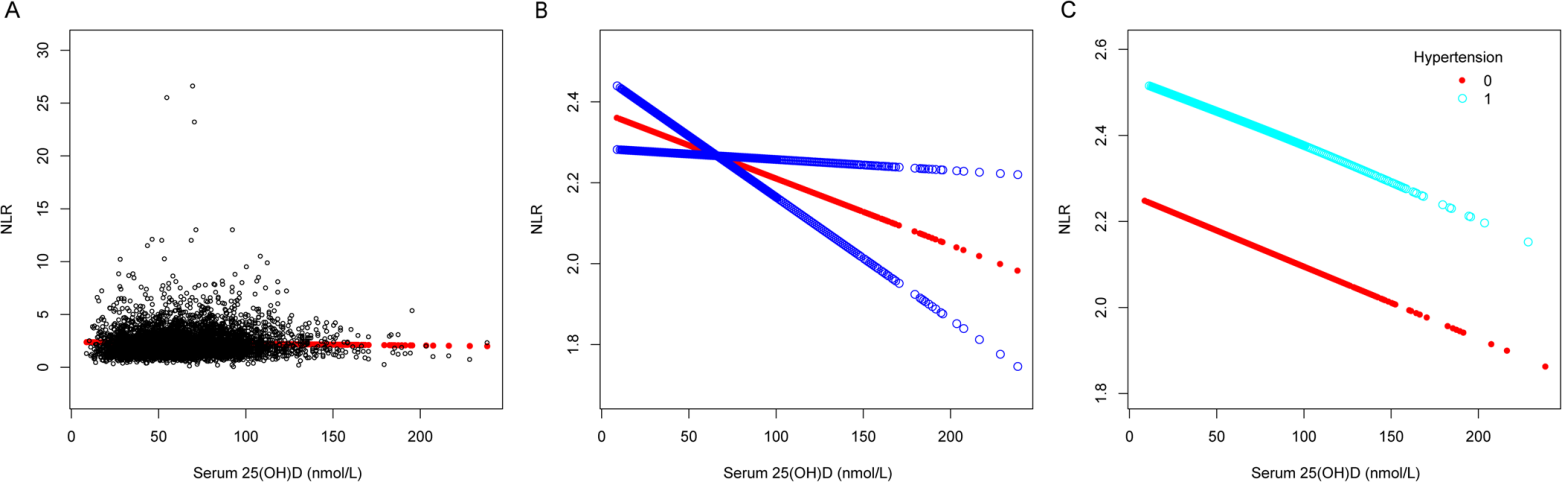
The association between serum 25(OH)D (nmol/L) and NLR. (A) Each black point represents a sample. (B) The smooth curve fit between the variables is represented by a solid red line. The blue bands indicate the 95% confidence interval (CI) of the fit. (C) Stratified by hypertension. The correlation coefficients for (B, C) are shown in the respective figures. Age, protein intake, BMI, alcohol intake, poverty income ratio, dietary fiber intake, ALT and AST levels, total blood calcium, triglyceride levels, sex, race, hypertension, diabetes, and smoking status were adjusted ([C] was not adjusted by hypertension).

**Figure 4:**
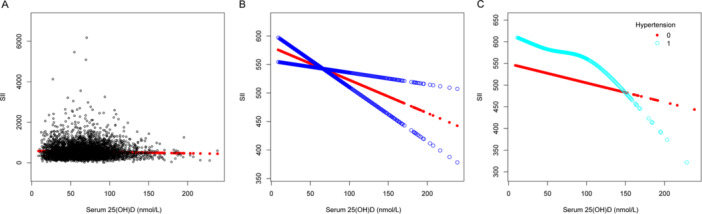
The association between serum 25(OH)D (nmol/L) and SII. (A) Each black point represents a sample. (B) The smooth curve fit between the variables is represented by a solid red line. The blue bands indicate the 95% confidence interval (CI) of the fit. (C) Stratified by hypertension. The correlation coefficients for (B, C) are shown in the respective figures. Age, protein intake, BMI, alcohol intake, poverty income ratio, dietary fiber intake, ALT and AST levels, total blood calcium, triglyceride levels, sex, race, hypertension, diabetes, and smoking status were adjusted ([C] was not adjusted by hypertension).

**Figure 5:**
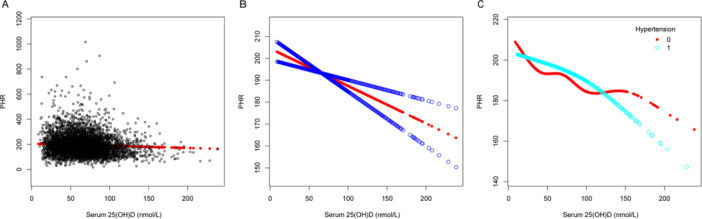
The association between serum 25(OH)D (nmol/L) and PHR. (A) Each black point represents a sample. (B) The smooth curve fit between the variables is represented by a solid red line. The blue bands indicate the 95% confidence interval (CI) of the fit. (C) Stratified by hypertension. The correlation coefficients for (B, C) are shown in the respective figures. Age, protein intake, BMI, alcohol intake, poverty income ratio, dietary fiber intake, ALT and AST levels, total blood calcium, triglyceride levels, sex, race, hypertension, diabetes, and smoking status were adjusted ([C] was not adjusted by hypertension).

**Figure 6:**
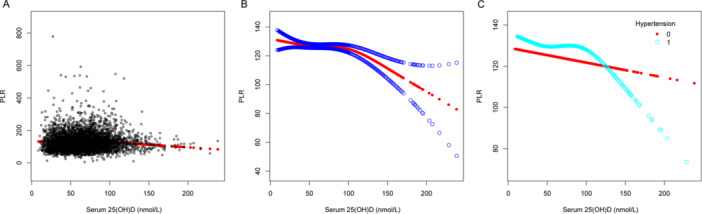
The association between serum 25(OH)D (nmol/L) and PLR. (A) Each black point represents a sample. (B) The smooth curve fit between the variables is represented by a solid red line. The blue bands indicate the 95% confidence interval (CI) of the fit. (C) Stratified by hypertension. The correlation coefficients for (B, C) are shown in the respective figures. Age, protein intake, BMI, alcohol intake, poverty income ratio, dietary fiber intake, ALT and AST levels, total blood calcium, triglyceride levels, sex, race, hypertension, diabetes, and smoking status were adjusted ([C] was not adjusted by hypertension).

Multivariable linear regression models were utilized to demonstrate the association involving serum 25(OH)D levels and several inflammatory indicators: NHR, NLR, SII, SIRI, PHR, and PLR. Model 1 had no covariate adjustments, while Model 2 included adjustments for age, sex, and race. After fully adjusting for age, sex, race, BMI, dietary fiber intake, alcohol intake, protein intake, poverty income ratio, ALT level, AST level, total blood calcium, triglyceride level, hypertension, diabetes, and smoking status in Model 3, we observed that serum 25(OH)D concentrations were negatively correlated with NHR (*β* = −0.003, 95% CI: −0.005 to −0.001, detailed in Table 2), NLR (*β* = −0.002, 95% CI: −0.003 to 0.000, detailed in Table 3), SII (*β* = −0.579, 95% CI: −0.954 to −0.205, detailed in Table 4), PHR (*β* = −0.171, 95% CI: −0.249 to −0.093, detailed in Table 5), and PLR (*β* = −0.096, 95% CI: −0.051 to −0.040, detailed in Table 6), but no association was observed with SIRI (*β* = −0.001, 95% CI: −0.002 to 0.000, detailed in Table 7). Different quartile groups of serum 25(OH)D exhibited a significant trend in relation to PHR and NHR (*p* < 0.05). After conducting subgroup analyses for hypertension and diabetes, we observed that serum 25(OH)D concentrations were negatively correlated with NHR, NLR, SII, SIRI, PHR, and PLR in the hypertensive population. Similarly, a significant negative link emerged between serum 25(OH)D levels and NHR, SII, PHR, and PLR in the nondiabetic population. To characterize the nonlinear relationships between serum 25(OH)D and NHR, NLR, SII, PHR, and PLR, we employed smooth curve fittings and generalized additive models, as detailed in Figures 2, 3, 4, 5, and 6, respectively. Subgroup analysis revealed inverted U‐shaped and U‐shaped associations between serum 25(OH)D concentrations and PLR, PHR, and SII in the hypertensive population. Furthermore, the corresponding inflection points for these relationships were calculated and are detailed in Table 8. For the hypertensive population, the specific inflection points after analysis are as follows: PLR at 103 nmol/L, PHR at 103 nmol/L, and SII at 105 nmol/L. When serum 25(OH)D concentrations remained above 103 nmol/L, each 1 nmol/L rise in serum 25(OH)D led to a statistically significant reduction of 0.518 in PLR and a reduction of 0.553 in PHR. However, this relationship did not reach statistical significance when serum 25(OH)D concentrations were below 103 nmol/L. For SII, a similar pattern was evident, showing a significant reduction of 2.485 with each 1 nmol/L increase in serum 25(OH)D levels above 105 nmol/L, although this trend was not observed when levels were below 105 nmol/L.

**Table 2:** Multivariable linear regression for the association between serum 25(OH)D (nmol/L) and NHR.

	Model 1	Model 2	Model 3
	*β* (95% CI)	*β* (95% CI)	*β* (95% CI)
**Serum 25(OH)D (nmol/L)**	−0.006 (−0.008, −0.004)*	−0.009 (−0.011, −0.007)*	−0.003 (−0.005, −0.001)*
**Quintiles of serum 25(OH)D**			
Lowest quintile (28.32–45.9 nmol/L)	Reference	Reference	Reference
Q2 (46.0–63.1 nmol/L)	0.165 (0.014, 0.315)*	−0.093 (−0.244, 0.058)	0.020 (−0.114, 0.153)
Q3 (63.2–81.4 nmol/L)	−0.117 (−0.267, 0.034)	−0.437 (−0.591, −0.283)*	−0.179 (−0.316, −0.042)*
Q4 (81.5–238 nmol/L)	−0.340 (−0.490, −0.189)*	−0.633 (−0.792, −0.474)*	−0.216 (−0.360, −0.073)*
*p* for trend	< 0.001	< 0.001	0.011
**Stratified by diabetes** a			
Yes	−0.002 (−0.007, 0.003)	−0.004 (−0.009, 0.001)	−0.001 (−0.005, 0.004)
No	−0.007 (−0.009, −0.005)*	−0.009 (−0.012, −0.007)*	−0.003 (−0.005, −0.001)*
**Stratified by hypertension** b			
Yes	−0.007 (−0.010, −0.004)*	−0.009 (−0.012, −0.006)*	−0.004 (−0.007, −0.001)*
No	−0.006 (−0.008, −0.003)*	−0.009 (−0.011, −0.006)*	−0.002 (−0.004, 0.000)

*Note:* Model 1: no covariates were adjusted. Model 2: age, gender and race were adjusted. Model 3: age, gender, race, poverty income ratio, BMI, alcohol intake, protein intake, dietary fiber intake, ALT level, AST level, total blood calcium, triglyceride level, hypertension, diabetes, smoking status were adjusted.

* *p* < 0.05.

^a^ All models were not adjusted by diabetes.

^b^ All models were not adjusted by hypertension.

**Table 3:** Multivariable linear regression for the association between serum 25(OH)D (nmol/L) and NLR.

	Model 1	Model 2	Model 3
	*β* (95% CI)	*β* (95% CI)	*β* (95% CI)
**Serum 25(OH)D (nmol/L)**	0.002 (0.001, 0.003)*	−0.002 (−0.004, −0.001)*	−0.002 (−0.003, −0.000)*
**Quintiles of serum 25(OH)D**			
Lowest quintile (28.32–45.9 nmol/L)	Reference	Reference	Reference
Q2 (46.0–63.1 nmol/L)	0.105 (0.004, 0.205)*	‐0.046 (−0.146, 0.053)	−0.023 (−0.122, 0.077)
Q3 (63.2–81.4 nmol/L)	0.119 (0.019, 0.219)*	‐0.127 (−0.229, −0.025)*	−0.081 (−0.183, 0.021)
Q4 (81.5–238 nmol/L)	0.192 (0.092, 0.292)*	‐0.163 (−0.268, −0.058)*	−0.105 (−0.211, 0.002)
*p* for trend	< 0.001	0.009	0.145
**Stratified by diabetes** a			
Yes	0.003 (‐0.002, 0.007)	−0.003 (−0.007, 0.002)	−0.003 (−0.007, 0.002)
No	0.002 (0.001, 0.003)*	−0.002 (−0.004, −0.001)*	−0.001 (−0.003, 0.000)
**Stratified by hypertension** b			
Yes	0.001 (−0.001, 0.004)	−0.003 (−0.006, −0.001)*	−0.003 (−0.005, −0.000)*
No	0.002 (0.001, 0.003)*	−0.002 (−0.003, −0.000)*	−0.001 (−0.002, 0.001)

*Note:* Model 1: no covariates were adjusted. Model 2: age, gender and race were adjusted. Model 3: age, gender, race, poverty income ratio, BMI, alcohol intake, protein intake, dietary fiber intake, ALT level, AST level, total blood calcium, triglyceride level, hypertension, diabetes, smoking status were adjusted.

* *p* < 0.05.

^a^ All models were not adjusted by diabetes.

^b^ All models were not adjusted by hypertension.

**Table 4:** Multivariable linear regression for the association between serum 25(OH)D (nmol/L) and SII.

	Model 1	Model 2	Model 3
	*β* (95% CI)	*β* (95% CI)	*β* (95% CI)
**Serum 25(OH)D (nmol/L)**	0.161 (−0.183, 0.506)	−0.812 (−1.178, −0.446)*	−0.579 (−0.954, −0.205)*
**Quintiles of serum 25(OH)D**			
Lowest quintile (28.32–45.9 nmol/L)	Reference	Reference	Reference
Q2 (46.0–63.1 nmol/L)	10.581 (−16.355, 37.517)	−17.088 (−44.199, 10.023)	−10.545 (−37.559, 16.469)
Q3 (63.2–81.4 nmol/L)	15.094 (−11.792, 41.979)	−33.310 (−60.981, −5.640)*	−21.645 (−49.450, 6.160)
Q4 (81.5–238 nmol/L)	20.070 (−6.826, 46.965)	‐52.458 (−81.026, −23.889)*	−34.680 (−63.720, −5.639)*
*p* for trend	0.026	0.005	0.125
**Stratified by diabetes** a			
Yes	0.228 (−0.820, 1.276)	−0.506 (−1.608, 0.596)	−0.421 (−1.550, 0.708)
No	0.126 (−0.232, 0.484)	−0.875 (−1.257, −0.493)*	−0.608 (−0.999, −0.216)*
**Stratified by hypertension** b			
Yes	−0.112 (−0.714, 0.490)	−1.106 (−1.740, −0.471)*	−0.945 (−1.593, −0.298)*
No	0.280 (−0.115, 0.676)	−0.558 (−0.981, −0.136)*	−0.215 (−0.648, 0.218)

*Note:* Model 1: no covariates were adjusted. Model 2: age, gender and race were adjusted. Model 3: age, gender, race, poverty income ratio, BMI, alcohol intake, protein intake, dietary fiber intake, ALT level, AST level, total blood calcium, triglyceride level, hypertension, diabetes, smoking status were adjusted.

* *p* < 0.05.

^a^ All models were not adjusted by diabetes.

^b^ All models were not adjusted by hypertension.

**Table 5:** Multivariable linear regression for the association between serum 25(OH)D (nmol/L) and PHR.

	Model 1	Model 2	Model 3
	*β* (95% CI)	*β* (95% CI)	*β* (95% CI)
**Serum 25(OH)D (nmol/L)**	−0.380 (−0.459, −0.301)*	−0.354 (−0.438, −0.270)*	−0.171 (−0.249, −0.093)*
**Quintiles of serum 25(OH)D**			
Lowest quintile (28.32–45.9 nmol/L)	Reference	Reference	Reference
Q2 (46.0–63.1 nmol/L)	2.346 (−3.828, 8.520)	−1.321 (−7.563, 4.921)	1.196 (−4.439, 6.831)
Q3 (63.2–81.4 nmol/L)	−6.997 (−13.159, ‐0.834)*	−9.853 (−16.223, −3.482)*	−3.077 (−8.877, 2.723)
Q4 (81.5–238 nmol/L)	−23.345 (−29.510, −17.180)*	−22.120 (−28.697, −15.542)*	‐8.790 (−14.848, −2.733)*
*p* for trend	< 0.001	< 0.001	0.006
**Stratified by diabetes** a			
Yes	−0.255 (−0.476, −0.034)*	−0.135 (−0.364, 0.093)	−0.015 (−0.238, 0.207)
No	−0.411 (−0.495, −0.327)*	−0.394 (−0.483, −0.304)*	−0.205 (−0.288, −0.123)*
**Stratified by hypertension** b			
Yes	−0.392 (−0.514, −0.270)*	−0.338 (−0.466, −0.210)*	−0.208 (−0.329, −0.087)*
No	−0.378 (−0.483, −0.274)*	−0.367 (−0.478, −0.256)*	−0.135 (−0.237, −0.032)*

*Note:* Model 1: no covariates were adjusted. Model 2: age, gender and race were adjusted. Model 3: age, gender, race, poverty income ratio, BMI, alcohol intake, protein intake, dietary fiber intake, ALT level, AST level, total blood calcium, triglyceride level, hypertension, diabetes, smoking status were adjusted.

* *p* < 0.05.

^a^ All models were not adjusted by diabetes.

^b^ All models were not adjusted by hypertension.

**Table 6:** Multivariable linear regression for the association between serum 25(OH)D (nmol/L) and PLR.

	Model 1	Model 2	Model 3
	*β* (95% CI)	*β* (95% CI)	*β* (95% CI)
**Serum 25(OH)D (nmol/L)**	0.060 (0.009, 0.111)*	−0.053 (−0.108, 0.001)*	−0.096 (−0.151, −0.040)*
**Quintiles of serum 25(OH)D**			
Lowest quintile (28.32–45.9 nmol/L)	Reference	Reference	Reference
Q2 (46.0–63.1 nmol/L)	1.109 (−2.894, 5.111)	−0.058 (−4.095, 3.978)	−1.058 (−5.070, 2.953)
Q3 (63.2–81.4 nmol/L)	5.238 (1.243, 9.233)*	1.734 (−2.386, 5.854)	−0.661 (−4.790, 3.468)
Q4 (81.5–238 nmol/L)	5.485 (1.488, 9.481)*	−2.051 (−6.305, 2.203)	−4.947 (−9.259, −0.634)*
*p* for trend	< 0.001	0.975	0.170
**Stratified by diabetes** a			
Yes	−0.007 (−0.154, 0.140)	−0.087 (−0.242, 0.068)	−0.108 (−0.265, 0.049)
No	0.073 (0.019, 0.127)*	−0.051 (−0.109, 0.007)	−0.095 (−0.154, −0.036)*
**Stratified by hypertension** b			
Yes	0.012 (−0.073, 0.098)	−0.114 (−0.204, −0.024)*	−0.142 (−0.233, −0.051)*
No	0.091 (0.029, 0.154)*	0.000 (−0.067, 0.067)	−0.047 (−0.116, 0.022)

*Note:* Model 1: no covariates were adjusted. Model 2: age, gender and race were adjusted. Model 3: age, gender, race, poverty income ratio, BMI, alcohol intake, protein intake, dietary fiber intake, ALT level, AST level, total blood calcium, triglyceride level, hypertension, diabetes, smoking status were adjusted.

* *p* < 0.05.

^a^ All models were not adjusted by diabetes.

^b^ All models were not adjusted by hypertension.

**Table 7:** Multivariable linear regression for the association between serum 25(OH)D (nmol/L) and SIRI.

	Model 1	Model 2	Model 3
	*β* (95% CI)	*β* (95% CI)	*β* (95% CI)
**Serum 25(OH)D (nmol/L)**	0.001 (0.000, 0.002)*	−0.002 (−0.003, −0.001)*	−0.001 (−0.002, 0.000)
**Quintiles of serum 25(OH)D**			
Lowest quintile (28.32−45.9 nmol/L)	Reference	Reference	Reference
Q2 (46.0–63.1 nmol/L)	−0.000 (−0.012, 0.011)	−0.006 (−0.017, 0.006)	−0.004 (−0.015, 0.008)
Q3 (63.2–81.4 nmol/L)	0.011 (0.001, 0.020)*	0.009 (−0.000, 0.018)	0.010 (0.001, 0.019)*
Q4 (81.5–238 nmol/L)	−0.000 (−0.003, 0.002)	−0.000 (−0.003, 0.002)	−0.000 (−0.003, 0.002)
P for trend	< 0.001	0.009	0.387
**Stratified by diabetes** a			
Yes	0.002 (−0.001, 0.005)	−0.001 (−0.005, 0.002)	−0.001 (−0.004, 0.002)
No	0.001 (−0.000, 0.002)	−0.002 (−0.003, −0.001)*	−0.001 (−0.002, 0.000)
**Stratified by hypertension** b			
Yes	0.001 (−0.001, 0.002)	−0.003 (−0.005, −0.001)	−0.002 (−0.004, −0.000)*
No	0.001 (0.000, 0.002)	−0.001 (−0.002, 0.000)	0.000 (‐0.001, 0.001)

*Note:* Model 1: no covariates were adjusted. Model 2: age, gender and race were adjusted. Model 3: age, gender, race, poverty income ratio, BMI, alcohol intake, protein intake, dietary fiber intake, ALT level, AST level, total blood calcium, triglyceride level, hypertension, diabetes, smoking status were adjusted.

* *p* < 0.05.

^a^ All models were not adjusted by diabetes.

^b^ All models were not adjusted by hypertension.

**Table 8:** Threshold effect analysis of serum 25(OH)D (nmol/L) and novel inflammatory markers, stratified by hypertension.

		Exposure: 25(OH)D (nmol/L)		
		< Infection point	> Infection point	Log
Outcomes	Infection point	Adjusted *β* (95%CI)	Adjusted *β* (95%CI)	Likelihood ratio
**Hypertensive individuals**				
PLR	103	−0.036 (−0.154, 0.082)	−0.518 (−0.800, −0.236)*	0.006
PHR	103	−0.111 (−0.268, 0.047)	−0.553 (−0.930, −0.177)*	0.057
SII	105	−0.548 (−1.376, 0.280)	−2.485 (−4.590, −0.379)*	0.130

*Note:* Age, gender, race, poverty income ratio, BMI, alcohol intake, protein intake, dietary fiber intake, ALT level, AST level, total blood calcium, triglyceride level, diabetes, and smoking status were adjusted.

* *p* < 0.05.

## 4 Discussion

Using the NHANES 2007–2018 data set, we analyzed the relationship between serum 25(OH)D and NHR, NLR, SII, SIRI, PHR, and PLR In the US adult population. Our findings revealed a negative relationship between serum 25(OH)D and NHR, NLR, SII, PHR, and PLR, while no association was observed with SIRI. When stratified by hypertension and diabetes, 25(OH)D levels exhibited a negative association with NHR, NLR, SII, SIRI, PHR, and PLR in the hypertensive group. Whereas, in the non‐hypertensive group, only PHR maintained this relationship. In the nondiabetic group, negative correlations were found between serum 25(OH)D concentrations and NHR, PHR, PLR, and SII, whereas in the diabetic group, no correlation was detected between these novel inflammatory markers and 25(OH)D levels.

Similar associations between serum 25(OH)D levels and novel inflammatory markers have been observed across different populations. For instance, a study by Kara AV et al. [24] showed that in hemodialysis patients, there was a negative correlation between NLR and PLR and serum 25(OH)D levels. Similarly, the study by Okuyan et al. demonstrated that vitamin D deficiency was associated with elevated levels of inflammatory markers (NLR and PLR) [25]. The findings mentioned above align with our results in the US adult population. Conversely, research by Dziedzic EA et al. [26] investigating the link between NLR and blood 25(OH)D in postmyocardial infarction patients found that NLR did not correlate with blood 25(OH)D. However, Dziedzic EA et al. also noted that serum 25(OH)D levels were negatively correlated with both SII and SIRI in individuals diagnosed with acute coronary syndrome [27]. Despite this, our findings indicate a significant negative association between SII and serum 25(OH)D concentrations in the US adult population, with no correlation with SIRI. To date, no research has investigated the association between PHR or NHR and serum 25(OH)D. This study is the first to report a notable inverse relationship between serum 25(OH)D levels and PHR or NHR. Taken together, findings from previous research and our current study suggest that adequate vitamin D supplementation may help mitigate systemic inflammation. Future studies with rigorous design are needed to further explore the bidirectional relationship between these inflammatory markers and 25(OH)D, as well as their potential role as predictors of vitamin D levels. Specifically, future research could employ longitudinal studies or randomized controlled trials to clarify the causal relationship between 25(OH)D levels and specific inflammatory markers. Additionally, predictive models incorporating inflammatory markers and vitamin D levels could be developed to evaluate their practical utility in early disease screening and prevention.

Serum 25‐(OH)D levels are frequently utilized to assess the status of vitamin D in the serum. Several mechanisms may underlie the impact of serum vitamin D on novel inflammatory markers. Vitamin D primarily affects monocytes among immune cells by preventing their transformation into dendritic cells. Reduced serum vitamin D levels contribute to the cytolytic and pro‐inflammatory characteristics observed in monocytes. Similarly, in neutrophils, vitamin D reduces adhesion and aggregation, leading to impaired migration, reduced synthesis of leukotriene B4, elevated ROS levels, and increased production of pro‐inflammatory cytokines [27]. Vitamin D receptors are thought to produce anti‐thrombotic effects. The expression of vitamin D receptors can be reduced by metabolically activated 1,25‐dihydroxyvitamin D. Conversely, a deficiency in vitamin D promotes the maturation of megakaryocytes and leads to higher thrombocyte counts [28]. Additionally, vitamin D disrupts the proliferation and differentiation of B cells into plasma cells that secrete antibodies [29]. Low serum vitamin D levels lead to decreased paraoxonase 1 activity, shifting high‐density lipoprotein from anti‐inflammatory to pro‐inflammatory molecules. Diminished paraoxonase activity exacerbates oxidative damage to lymphocyte DNA, thereby affecting lymphocyte counts [30]. Further laboratory studies are needed to explore the specific mechanisms by which vitamin D affects various blood cells.

Our study is a cross‐sectional study, so it also has some advantages and disadvantages. Firstly, it is a large‐scale survey that includes a comprehensive data set of serum 25(OH)D concentrations, NHR, NLR, SII, SIRI, PHR, and PLR among a nationally representative sample of American adults (*n* = 5308). Secondly, this extensive sample size allows for detailed subgroup analyses, facilitating a deeper exploration of the unique links between blood 25(OH)D concentrations and these indicators across different diseases. Nevertheless, there are some limitations in this study: (i) we observed an association between serum 25(OH)D and inflammatory markers, due to the limitations of the cross‐sectional design, we cannot infer causality; (ii) the findings could be influenced by seasonal fluctuations, particularly during periods of low sunlight exposure; (iii) inflammation‐related dietary and lifestyle factors, including vitamin D, fat, and calcium intake, as well as smoking, alcohol consumption, and physical inactivity, may confound the results of this study.

## 5 Conclusion

In summary, blood 25(OH)D demonstrated negative correlations with NHR, NLR, SII, PHR, and PLR among the US adult population. However, no significant association was found with SIRI. When the data were stratified based on hypertension and diabetes, serum 25(OH)D showed negative correlations with NHR, NLR, SII, SIRI, PHR, and PLR in the hypertensive group. In the non‐hypertensive group, only PHR maintained this relationship. Among the nondiabetic group, negative correlations were noted between serum 25(OH)D and NHR, PHR, PLR, and SII, whereas no correlations were found with these novel inflammatory markers in the diabetic group.

## Author Contributions

Hang Zhao contributed to data collection, analysis, and drafting the initial manuscript. Yangyang Zhao contributed to drafting and reviewing the initial manuscript. Yini Fang was responsible for refining the manuscript. Wenjing Zhang was responsible for creating the tables. Weibang Zhou was responsible for creating the figures. Jiecheng Peng was responsible for selecting topics, guiding study design, and reviewing the manuscript. All authors contributed to this work and approved it for submission.

## Ethics Statement

This study was conducted in accordance with the principles of the Declaration of Helsinki. The research involving human participants was reviewed and approved by the Institutional Review Board of NCHS.

## Consent

All individual participants in the study provided informed consent.

## Conflicts of Interest

The author states that this study was conducted without any commercial or financial relationships that might be perceived as potential conflicts of interest.

## Data Availability

In this study, publicly available datasets were analyzed, which can be accessed at http://www.cdc.gov/nchs/nhanes/.
